# Genetically engineered pair of cells for serological testing and its application for SARS‐CoV‐2

**DOI:** 10.1002/btm2.10508

**Published:** 2023-03-24

**Authors:** Marvin A. Ssemadaali, Juan Arredondo, Elise A. Buser, Sherri Newmyer, Harikrishnan Radhakrishnan, Harold S. Javitz, Satya Dandekar, Parijat Bhatnagar

**Affiliations:** ^1^ Biosciences Division SRI International Menlo Park California 94025 United States; ^2^ Medical Microbiology and Immunology University of California Davis Davis California 95616 United States; ^3^ Education Division SRI International Menlo Park California 94025 United States

**Keywords:** cell engineering, cell‐based sensors, COVID‐19 diagnostics, serology test, synthetic biology

## Abstract

We have developed a serology test platform for identifying individuals with prior exposure to specific viral infections and provide data to help reduce public health risks. The serology test composed of a pair of cell lines engineered to express either a viral envelop protein (Target Cell) or a receptor to recognize the Fc region of an antibody (Reporter Cell), that is, *Diagnostic‐Cell‐Complex (DxCell‐Complex)*. The formation of an immune synapse, facilitated by the analyte antibody, resulted into a dual‐reporter protein expression by the Reporter Cell. We validated it with human serum with confirmed history of severe acute respiratory syndrome coronavirus 2 (SARS‐CoV‐2) infection. No signal amplification steps were necessary. The DxCell‐Complex quantitatively detected the target‐specific immunoglobulin G (IgG) within 1 h. Validation with clinical human serum containing SARS‐CoV‐2 IgG antibodies confirmed 97.04% sensitivity and 93.33% specificity. The platform can be redirected against other antibodies. Self‐replication and activation‐induced cell signaling, two attributes of the cell, will enable rapid and cost‐effective manufacturing and its operation in healthcare facilities without requiring time‐consuming signal amplification steps.

## 1 INTRODUCTION

Since its emergence in late 2019, severe acute respiratory syndrome coronavirus 2 (SARS‐CoV‐2) has continued to spread widely and cause devasting global economic losses.^1^ Pre‐symptomatic and asymptomatic viral transmission is responsible for the rapid spread that escapes surveillance strategies.^2^, ^3^, ^4^, ^5^ Successful control of the pandemic has mainly been through restricted travel, social distancing, use of facemasks, and vaccines. However, reopening economies are generating a growing interest in a widely accessible and cost‐effective serology test able to screen individuals for the presence of antibodies and measure their anti‐viral immunity.^6^

Our study reports a novel serology test platform, the antibody‐specific *Diagnostic‐Cell‐Complex* (*DxCell‐Complex*), that can be used to identify individuals harboring antibodies against specific viral antigens. We validated this test platform by detecting antibodies against SARS‐CoV‐2 from clinical specimens. Importantly, this platform can be quickly redirected to identify antibodies against any virus. This technology is based on a T cell's ability to regulate its activation cascade upon formation of the immune synapse with the antigen‐presenting cell and our ability to engineer the two cell types that compose the DxCell‐Complex, a T‐cell‐based Reporter Cell^7^, ^8^, ^9^ and an antigen‐presenting Target Cell.^10^

The Target Cell is a fast‐growing clone of an easy‐to‐transfect cell line derived from the kidney cells of a human embryo (HEK293T/17) that has been genetically engineered to stably display the spike glycoproteins (Sgp) of the SARS‐CoV‐2 virus. The sequence of Sgp can be exchanged to present any other viral antigenic biomarker. The Reporter Cell is a fast‐growing, immortalized human T‐acute lymphoblastic leukemia cell line (Jurkat cells, doubling time ~20 h^11^) genetically engineered to upregulate a dual reporter system that exhibits fluorescence (green fluorescent protein, GFP) and bioluminescence (NanoLuc or Nluc) on recognizing the Fc region of an Sgp‐specific immunoglobulin G (IgG) bound to Sgp on the Target Cell.

**FIGURE 1:**
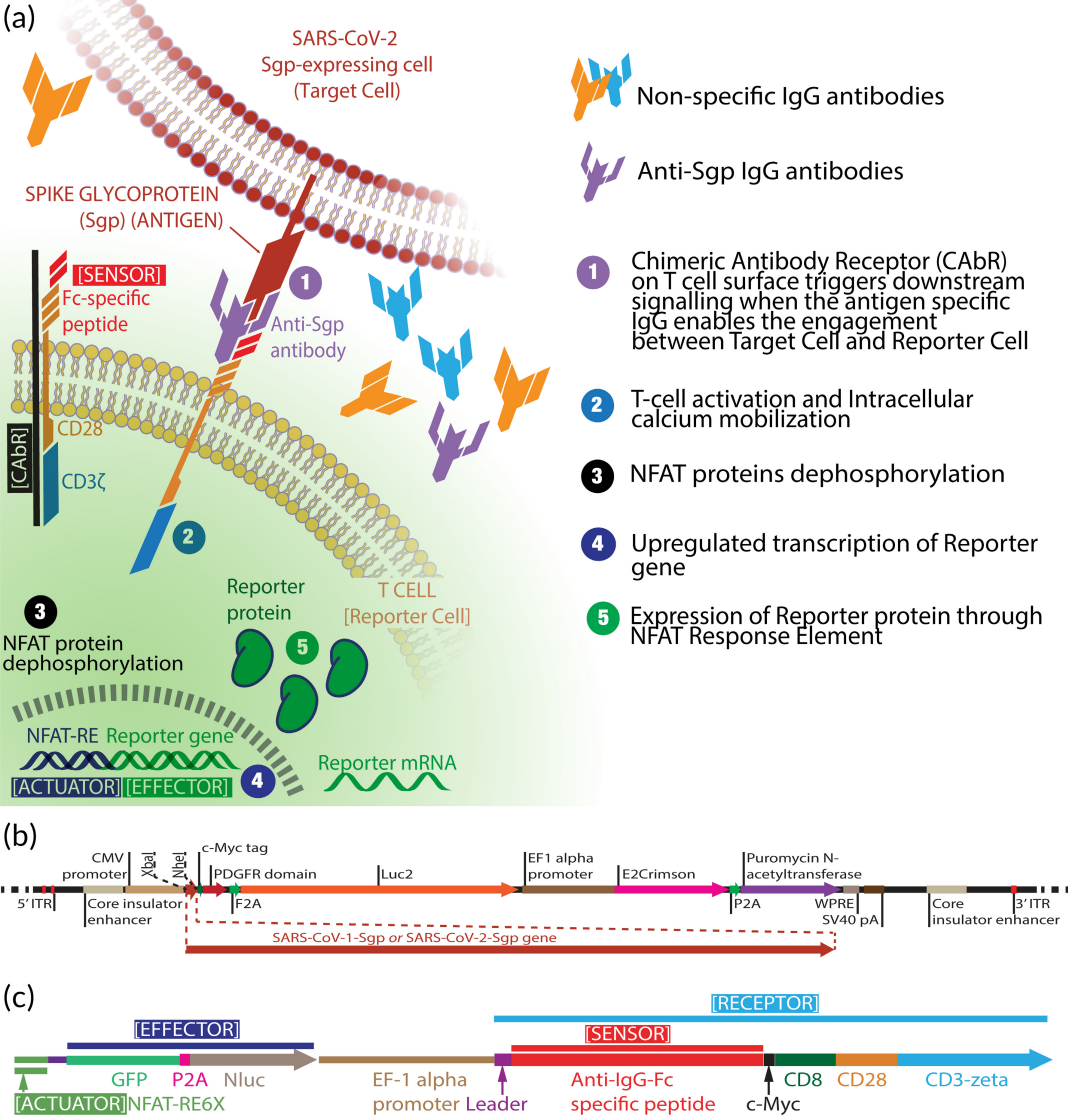
Schematic of the DxCell‐Complex and plasmid constructs. (a) Mechanism of action for the DxCell‐Complex platform. (b) Gene insert in the PiggyBac plasmid backbone for engineering the Target Cell with spike glycoprotein (contains S1 and S2 subunits). (c) Gene insert for engineering the Reporter Cell.

The schematic in Figure 1a illustrates how the DxCell‐Complex detects the Anti‐Sgp IgG antibodies. The Target Cell is composed of HEK293T/17 as the cellular chassis engineered to stably express the viral Sgp on its surface. The Sgp insert was sub‐cloned into the PiggyBac system backbone^10^ and was then used for stable integration (Figure 1b). This approach transforms the DxCell‐Complex serology test technology into a broadly applicable platform because the Sgp sequence can be exchanged for any antigenic biomarker on the Target Cell, thereby redirecting the specificity of the complex toward the antibody of interest. We used this strategy to develop the DxCell‐Complex with specificity for SARS‐CoV‐2 or SARS‐CoV‐1. Figure 1c details the gene insert^7^, ^8^ that was used with our lentivirus transduction process^9^ to generate the Reporter Cell with specificity toward the Fc region of IgG antibodies. The Reporter Cell is encoded with an artificial cell‐signaling pathway with the following three domains: the *Chimeric Antibody Receptor (CAbR)*, a transmembrane molecule that includes an extracellular Fc‐region binding portion (*Sensor*) at its distal end and mobilizes; the *Actuator*, the T‐cell's activation machinery that upregulates the *Effector*, a dual reporter system (GFP‐2A‐Nluc) upregulated when the antibody is also bound to the Sgp on the Target Cell through its Fab portion.

## 2 RESULTS

To illustrate the function of the antibody‐specific DxCell‐Complex for detecting the presence of virus‐specific antibodies and identifying individuals with antibodies against viral infections, we transformed the parental HEK293T/17 cell line into two different types of Target Cells: (1) SARS‐CoV‐1‐Sgp cells and (2) SARS‐CoV‐2‐Sgp cells, which constitutively and stably express the Sgp from SARS‐CoV‐1 and SARS‐CoV‐2, respectively. The Reporter Cell, on the other hand, was engineered to express GFP and Nluc transgenes as activation‐inducible reporters (linked through a self‐cleavable peptide linker) to quantitatively respond to the intensity of the stimulus. The Sensor portion (part of the CAbR) of the Reporter Cell comprises the sequence of the Fc‐region‐binding domain from the bacterial Protein A (zz‐domain^12^, ^13^; GenBank: M74186). The formation of immune synapse, assisted via the analyte antibody (see schematic in the Figure 1a), results in transcriptional regulation of the reporter system (Effector) through intracellular calcium rise^14^ for quantitative assessment of the infection. To demonstrate the functionality of the DxCell‐Complex and its ability to identify different antibody (IgG) types, we conducted parallel validation assays using IgG antibodies with specificity for both SARS‐CoV‐1 and SARS‐CoV‐2; the assays required incubation with the two different Target Cells, SARS‐CoV‐1‐Sgp cell and SARS‐CoV‐2‐Sgp cell, respectively. Isotype IgG against the envelop glycoprotein of the West Nile Virus (Anti‐WNV IgG) was used as a negative control in all panels and at the concentration similar to that of the analyte antibody.

**FIGURE 2:**
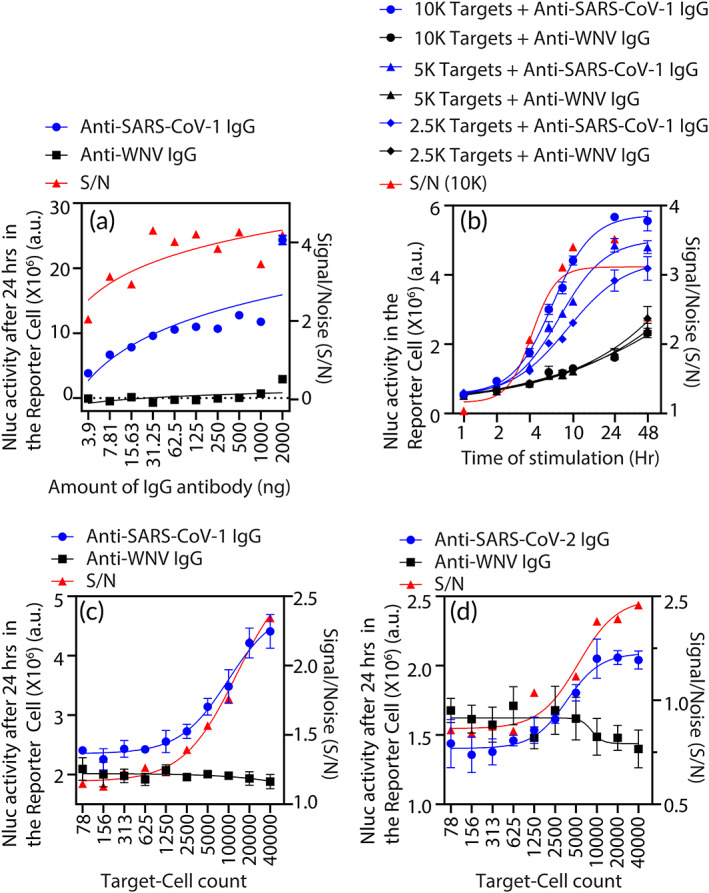
Development of method for using the DxCell‐Complex. The Reporter Cell was incubated with the Target Cell to form the DxCell‐Complex in presence of Target‐Cell specific IgG antibody. The Nluc activity in the Reporter Cell (12,500 cells) (a) is proportional to the Anti‐SARS‐CoV‐1 IgG antibody titer; (b) increased with respect to time; (c) increased proportionally to the number of engineered target SARS‐CoV‐1‐Sgp cells; (d) increased proportionally to the number of engineered target SARS‐CoV‐2‐Sgp cells. A total of 10,000 Target Cells were used for (a) and (b) and were varied in (c) and (d) as represented along the *x*‐axis. In all experiments, Anti‐WNV‐Egp antibody was used as a negative control. Nluc activity for all observations (a–d) was measured using *n* = 4; error bars indicate ±1 SD. IgG, immunoglobulin G; SARS‐CoV, severe acute respiratory syndrome coronavirus; Sgp, spike glycoprotein; WNV‐Egp, West Nile virus envelop glycoprotein.

Figure 2a demonstrates that, in addition to the qualitative detection of the infection, the Reporter (Nluc) activity of the DxCell‐Complex also provides a quantitative measure proportional to the antibody titer. The signal‐to‐noise ratio (S/N), defined in the figure legend, quantifies the sensitivity of the DxCell‐Complex. The S/N was ~2 at low IgG antibody titer (~4 ng) of the SARS‐CoV‐1‐specific IgG (*p* < 0.0001); the Reporter Cell to Target Cell ratio (R:T) = 1.25:1 and logarithmically increases to ~4 at higher titers, a generally reported observation in serology tests.^15^ The data in Figure 2b illustrate the reporter kinetics of the DxCell‐Complex upon engaging SARS‐CoV‐1‐specific IgG and compared to the isotype control antibodies. The DxCell‐Complex, with serially diluted Target Cells (10,000, 5000, and 2500 SARS‐CoV‐1‐Sgp cells) was used. The Nluc activity from Reporter Cell was detected as early as 2 h (S/N = ~1.4, *p* < 0.002) when a 10,000 Target Cells were used, and it increased steadily for the duration of the assay (at 48 h, *p* < 0.0001). The wide time window for the readout will assist mass screening efforts by accommodating a large test‐sample size. A representative S/N curve is included for 10,000 SARS‐CoV‐1‐Sgp cells. Additionally, while the signal from the Reporter Cell was high in proportion to the higher Target Cell count, the kinetics of the test were faster at a lower Target Cell count. At 1 h, a statistically significant increase in the signal was observed for 2500 targets (S/N = ~1.16, *p* < 0.005) and 5000 targets (S/N = ~1.14, *p* < 0.005), compared to 10,000 Target Cells (S/N = ~1.04, *p* = 0.16). These data validate our previous observations in context of the Nluc reporter activity of similarly engineered cells being proportional to the disease burden.^7^, ^9^ Figure 2c,d demonstrates the platform nature of the DxCell‐Complex and the effect of the Target Cell count on the sensitivity of the test. The data show that, when using the same amount of target‐specific IgG antibody, the Reporter‐Cell activity increases proportionately to the number of Target Cells. On the other hand, the signal from the DxCell‐Complex when incubated with the isotype‐control antibody did not increase. Figure 2c uses the SARS‐CoV‐1‐Sgp cells as Target Cells and detects SARS‐CoV‐1‐specific IgG; the signal was statistically higher compared to isotype control (*p* < 0.02 at all R:T < 20:1). Figure 2d uses the SARS‐CoV‐2‐Sgp cells to detect SARS‐CoV‐2‐specific IgG; signal in this case was statistically higher at all R:T < 1.25:1 (*p* < 0.002), compared to the isotype control. We note that although the trend in Figure 2b,c is preserved and supports the reproducibility of the assay, the signal intensity at certain points is different for the same number of Target Cells and Reporter Cells. While this may be due to difference in the growth phase of the cells and could be further investigated, we focused our efforts on the next stage of validating our technology using clinical specimens.

To characterize the performance of the above serology test platform for detecting SARS‐CoV‐2 IgG antibodies, we used a commercially available panel of serum samples from 10 COVID‐19 patients and 10 negative controls. All specimens were previously examined using the Abbott Architect IgG assay for SARS‐CoV‐2 IgG antibodies. Similar procedures, as reported in Figure 2, were employed to create the DxCell‐Complex, and the results were assessed for sensitivity and specificity. Patient sera was initially serially diluted (1:50, 1:100, 1:200) in the cell‐culture media to determine the optimal dilution for improved sensitivity (Figure S1). The dilution of 1:200 (*p* < 0.05) was selected for use in subsequent assays and can be further optimized for improved performance.

**FIGURE 3:**
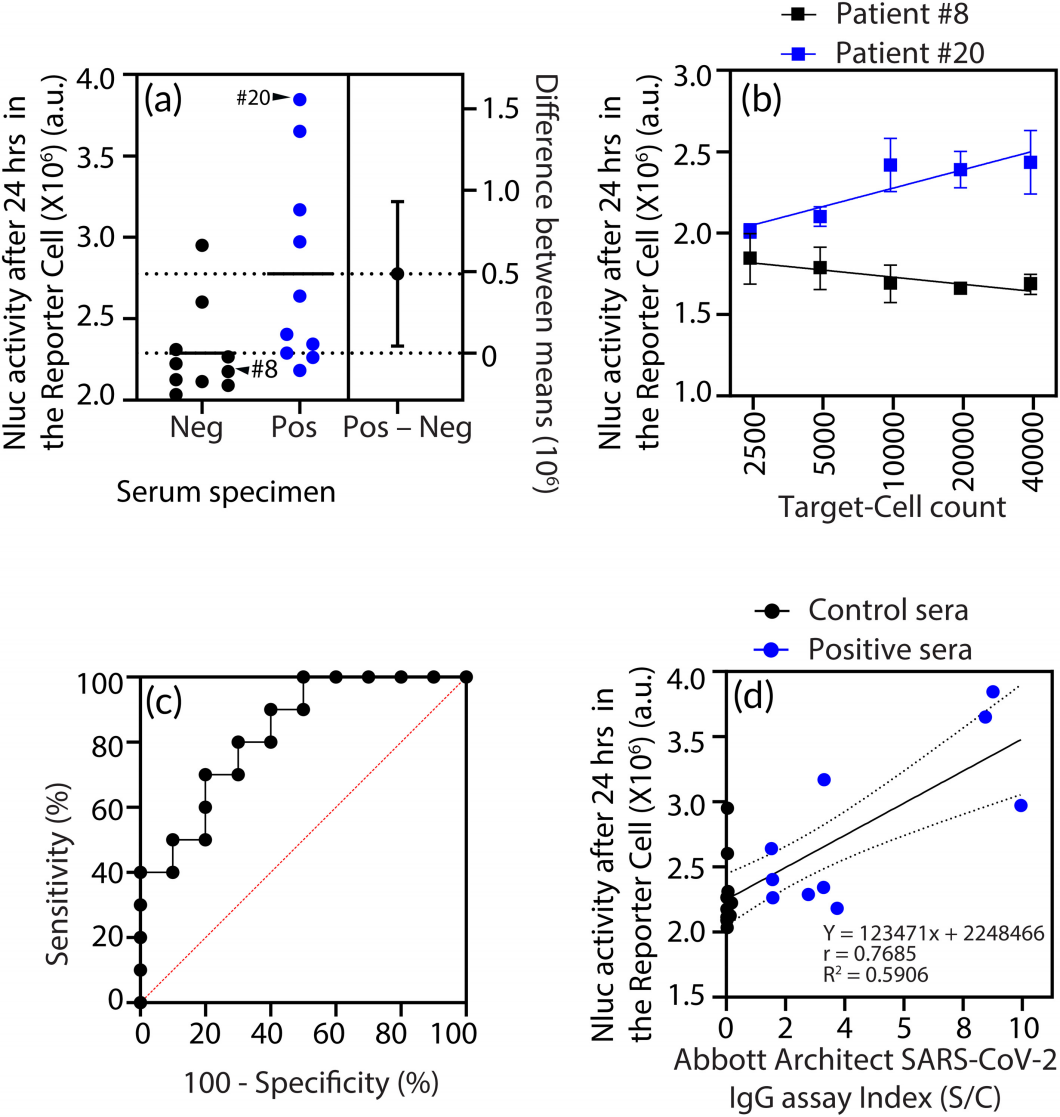
Characterization of the DxCell‐Complex serology test using commercial serum panel. (a) An estimation plot shows that the DxCell‐Complex differentiates (*p* < 0.05) between patient sera (positive, *n* = 10) and control sera (negative, *n* = 10) samples (1:200 sera dilution; 12,500 Reporter Cells; 10,000 target SARS‐CoV‐2‐Sgp cells; 24 h). The horizontal lines indicate the mean values for the respective groups. The *p* value was calculated using an unpaired two‐tailed Student's *t*‐test. (b) The Nluc activity in the Reporter Cell (12,500 cells) increased proportionally to the target SARS‐CoV‐2‐Sgp cells with specimen #20 (positive) serum but not with specimen #8 (negative) serum. (c) ROC analysis of the DxCell‐Complex, using the Abbott Architect IgG assay as the gold standard, with an AUC value of 0.830. (d) Correlation analysis of 20 SARS‐CoV‐2 sera panels with different levels of SARS‐CoV‐2 IgG antibodies analyzed by the Abbott Architect IgG assay versus the DxCell‐Complex. Comparison between results was performed using Pearson's correlation coefficient and simple linear regression analyses. Mean Nluc activity values of DxCell‐Complex are plotted on the *y*‐axis, while index values of the Abbott Architect IgG assay are shown on the *x*‐axis. Statistical significance was calculated using the two‐tailed test. The dashed lines indicate the standard deviations of the linear regression plots. Analysis in (c) and (d) is based on the data values obtained in (a). Nluc activity for all observations (a–d) was measured using *n* = 4; error bars indicate ±1 SD. AUC, area under the curve; IgG, immunoglobulin G; ROC, receiver operating characteristics; SARS‐CoV, severe acute respiratory syndrome coronavirus; Sgp, spike glycoprotein.

The estimation plot in Figure 3a demonstrates the magnitude of the difference in means of two clinical specimen groups (positive or negative for the SARS‐CoV‐2 antibodies) as assessed by our serology test. At the 95% confidence interval, the difference between the two means is greater than zero, demonstrating statistical significance (*p* < 0.05) of the test. Figure 3b illustrates an approach in context of the DxCell‐Complex platform, which we introduced to differentiate between the seropositive specimens at a low antibody titer from the seronegative specimens and further reduce any potential of false positive signal. Based on our observation in Figure 2c,d, we reasoned that, unlike the seronegative specimen, incubation of the fixed quantity of seropositive specimen with serially diluted Target‐Cell count will proportionately reduce the stimulation of the Reporter Cell and show up as a slope of the Reporter Cell signal. We confirmed this approach by repeating the experiment using clinical specimens with serially diluted Target‐Cell count with results shown in Figure 3b. The presence of positive slope in the Reporter‐Cell signal from the DxCell‐Complex, when incubated with a seropositive specimen differentiates it from the seronegative specimen (*p* < 0.004 at all R:T ≤ 2.5:1) and offers to minimize the instances of false‐negatives and false‐positives. Note the difference in Nluc activity of the Reporter Cell, as presented in on the *y*‐axis of Figure 3a,b, may be due to the difference in the growth phase of the Reporter Cells.

To determine the robustness of the serology test based on the DxCell‐Complex and its performance in clinical‐sample testing, we used receiver operating characteristics (ROC) curve analysis, as shown in Figure 3c. Using the Abbott Architect IgG assay as the gold‐standard test, the ROC curve showed an area under the curve (AUC) value of 0.83 (*p* < 0.05). At a cut‐off value of 2,276,910 a.u., our serology assay showed an optimal sensitivity and specificity of 80% and 70%, respectively. Table S1 reports a complete analysis of the results,^16^ showing a positive predictive value of 72.7%, a negative predictive value of 77.8%, and a likelihood ratio of 2.667. Figure 3d demonstrates the linear relationship between results from our DxCell‐Complex serology test and results from the Abbott Architect IgG assay using the Pearson‐correlation analysis that produced a correlation coefficient (*r*) of 0.77. Linear regression analysis indicated the *R* ^2^ value of 0.59 [*F* (1, 18) = 25.96, (*p* < 0.0001)] when Reporter‐Cell activity from the DxCell‐Complex‐based serology test was plotted against the Abbott Architect IgG assay values.

**FIGURE 4:**
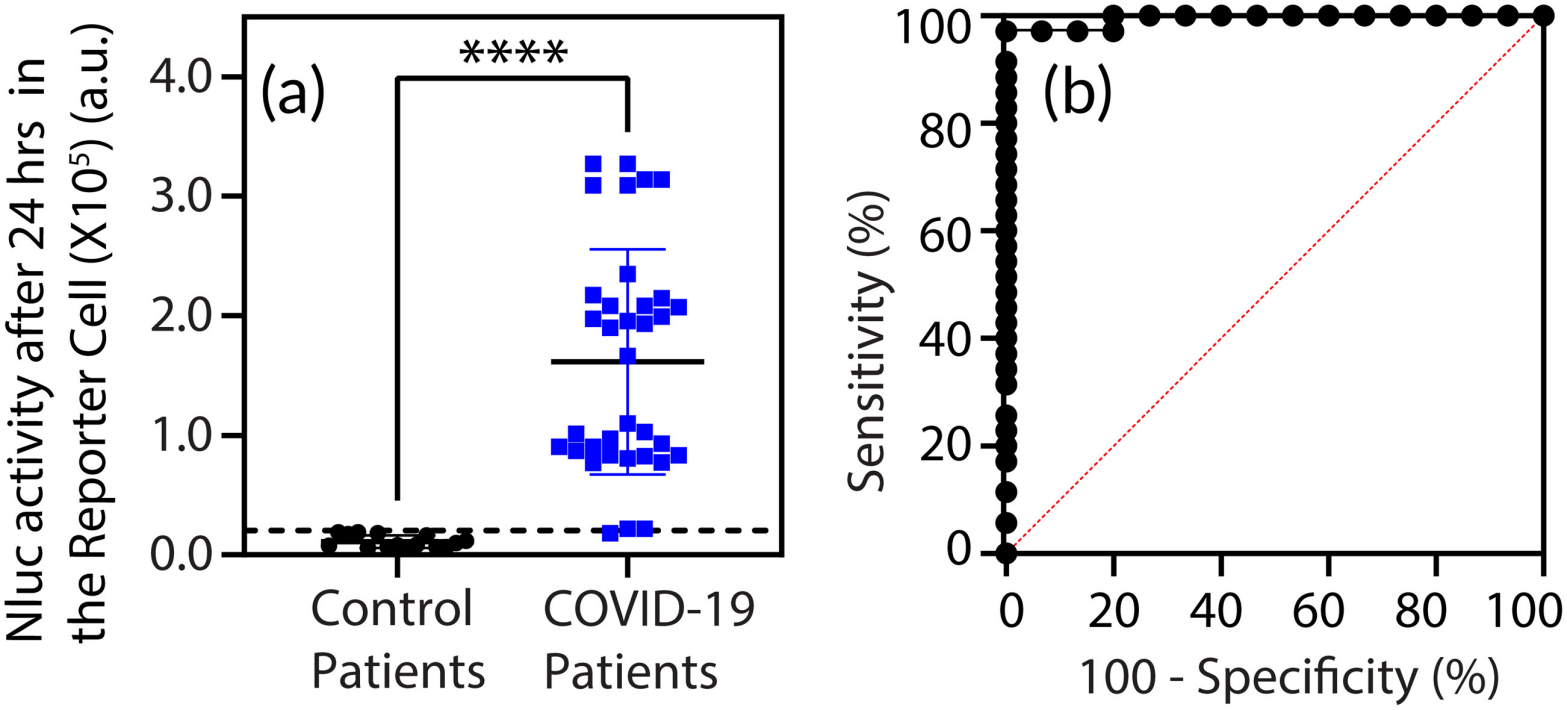
Clinical validation of the DxCell‐Complex for detecting COVID‐19 IgG antibodies in patients. (a) Scatter plot shows that the DxCell‐Complex significantly differentiates between control subjects and COVID‐19 patients (****p < 0.0001). The dotted horizontal line represents a positive cut‐off value based on the ROC analysis shown in (b). The *p* value was calculated using an unpaired two‐tailed Student's *t*‐test. Nluc activity for each serum sample was measured using *n* = 4; error bars indicate ±1 SD of the mean. (b) ROC analysis of the DxCell‐Complex, based on qPCR results, with an AUC value of 0.9941. Patient sera was diluted at 1:200, incubated with Reporter Cell (12,500 cells), washed, and incubated with target SARS‐CoV‐2‐Sgp cells (10,000 cells) for 24 h (COVID‐19 patients, *n* = 34; control patients, *n* = 15). Analysis in (b) is based on the data values obtained in (a). AUC, area under the curve; IgG, immunoglobulin G; ROC, receiver operating characteristics; SARS‐CoV, severe acute respiratory syndrome coronavirus; Sgp, spike glycoprotein.

To ensure reliability and reproducibility, Figure 4 illustrates the results from the serology test conducted on another set of 49 serum samples (control subjects = 15, COVID‐19 patients = 34) obtained from UC Davis Health. Serum samples were collected from individuals who were diagnosed for SARS‐CoV‐2 infection (males = 16, out of which 8 had recovered, age range of 25–77 years; females = 18, out of which 8 had recovered, age range of 22–74 years) based on the qPCR detection of SARS‐CoV‐2 RNA as well as seropositivity for SARS‐CoV‐2 IgG by ELISA. Serum samples from healthy SARS‐CoV‐2 seronegative individuals (males = 6, age range of 25–68 years; females = 9, age range of 23–65 years) were used as negative controls to test the specificity of the cell‐based assay. Figure 4a shows that our serology test detected Sgp‐IgG antibodies in individuals with prior exposure to SARS‐CoV‐2 virus compared to the control subjects (*p* < 0.0001). For the proof‐of‐principle studies, we assessed the signal from the DxCell‐Complex after 24 h. However, our results in Figure 2b show that the test can be significantly faster with the potential to inform on the seropositivity within 2 h. Based on the clinical status (qPCR result), the ROC curve analysis in Figure 4b further demonstrates the accuracy of the DxCell‐Complex serology assay with an AUC value of 0.9941 (*p* < 0.0001). At a cut‐off value of 18,824 a.u., our serology assay showed an optimal sensitivity of 97.04% and specificity of 93.33% for detecting anti‐Sgp IgG antibodies. Table S2 shows a contingency‐table analysis^16^ with a positive predictive value of 97.04%, a negative predictive value of 93.33%, and a likelihood ratio of 14.56. We anticipate that the improved performance of the DxCell‐Complex when testing the clinical samples in Figure 4, compared to when testing the commercially available panel of patient serum in Figure 3, was due to the heat‐inactivation treatment (65°C for 30 min) employed in Figure 3. While this heat treatment allowed safe handling of the specimens in our biosafety level 2 (BSL2) laboratory, it could have denatured the antibodies reducing the sensitivity and specificity of the DxCell‐Complex.

## 3 DISCUSSION

Although travel restrictions, social distancing, the use of facemasks, and vaccines have led to a decline in COVID‐19 infections, new COVID‐19 infections have continually decimated the global economy. During this public health crisis, roughly half of the world's population was forced to stay at home and the unemployment rate rose to 25%. To help managing this crisis, we recently reported on a cell‐based platform and used it for antigen testing.^17^ More efforts are still needed on developing the capacity for serology testing with improved accuracy^18^, ^19^, ^20^ that may assist with evaluating disease prevalence, the level of protective immunity, and the efficacy of the vaccination programs in the population.^6^ In this work, we have applied the DxCell platform for low‐cost and rapid serology testing that may help with informed public health policies leading to the re‐opening of global economies.

In this study, we used a cell‐engineering approach to develop a serology test for detecting the SARS‐CoV‐2‐Sgp‐specific IgG as a marker of disease seroprevalence. The DxCell‐Complex serology test was validated using clinical specimens with previously confirmed COVID‐19 infection and serology. Our data show that the performance characteristics of the DxCell‐Complex serology test could exceed the minimum requirements listed for serology tests undergoing FDA emergency use authorization submission.^21^ The DxCell‐Complex has the potential to identify individuals with recent or prior SARS‐CoV‐2 infections, and offers unique advantages by enabling: (1) cost‐effective mass‐testing capabilities in decentralized healthcare facilities and with easy sample preparation steps; (2) an easily transportable quantitative, self‐replicating diagnostic tool that works in a label‐free manner without signal amplification steps; (3) a practical approach for manufacturability; and (4) due to its modularity,^7^, ^8^ this cell‐based testing platform that can be transformed into a therapeutic tool by exchanging the Reporter protein with a therapeutic protein, as demonstrated by our group.^22^, ^23^

This work complements existing serological testing technologies^18^, ^20^, ^24^, ^25^ enabling informed public health decisions required to reopen economies. For example, it will enable identification of individuals who may have seroconverted due to vaccination or prior SARS‐CoV‐2 infection. These individuals can be cleared to care for others with COVID‐19 and return to in‐person employment. The test involves a simple, one‐step procedure that quantitatively informs on the antibody titer in seropositive individuals and can be undertaken in BSL2 settings at local healthcare facilities. This platform technology can also be redirected toward emerging COVID variants and other viruses by exchanging the viral envelop/spike protein in the Target Cells.

## 4 METHODS AND MATERIALS

No unexpected or unusually high safety hazards were encountered during the completion of this work.

### 4.1 Materials and reagents

Jurkat E6‐1 (ATCC, Cat #TIB‐152) cell line was maintained in complete RPMI media (RPMI1640 [Corning, Cat #10‐040‐CV], 10% heat‐inactivated fetal bovine serum or FBS [Sigma‐Aldrich, Cat #F2442‐500ML], and 1× penicillin–streptomycin solution [Corning, Cat #30‐002‐Cl]). HEK293T/17 cells (ATCC, Cat #CRL‐11268) cultured in complete DMEM (DMEM growth media [Corning, Cat #10‐013‐CV] supplemented with 10% heat‐inactivated FBS and 1× penicillin–streptomycin). Plasmids encoding different genetic payloads (transfer plasmids) were designed in SnapGene software (GSL Biotech LLC) and sub‐cloned into lentivirus vector plasmid (System Biosciences, Cat #CD510B‐1). Second generation packaging plasmids (psPAX2 [Cat #12260] and pMD2.G [Cat #12259]) were obtained from Didier Trono (Ecole Polytechnique Fédérale de Lausanne, Lausanne, Switzerland) through Addgene. pAdVAntage^TM^ was obtained from Promega (Cat #E1711). PiggyBac Transposase from Reference 26 was used. All plasmid preparation services (chemical synthesis of DNA insert sequences, sub‐cloning into respective vector backbones, and the amplification) were obtained from Epoch Life Science, Inc. (Missouri City, TX). For lentivirus production, plasmid transfections into parental HEK293T/17 cells were performed using Transporter 5 reagent (Polysciences, Inc., Cat #26008‐5). Polybrene (abm, Cat #G062) was used for lentivirus transductions into Jurkat cells. TransIT‐2020 transfection reagent (Mirus #MIR5400) was used to transfect and make stable Target Cells. Puromycin dihydrochloride (ThermoFisher Scientific, Cat# A1113803) was used for selecting stable cells. Anti‐SARS‐CoV‐1 Sgp monoclonal antibody or S230 (Absolute Antibody, Cat# Ab00268), Anti‐SARS‐CoV‐2 Sgp monoclonal antibody (SinoBiological, Cat# 40150‐R007), and Anti‐WNV envelop glycoprotein (WNV‐Egp) monoclonal antibody (SinoBiological, Cat# 40345‐MM03) were used for characterization experiments of the DxCell‐Complex. Nano‐Glo assay kit (Promega, Cat #N1120) was used to assess expression of the Nluc protein.

### 4.2 Source of COVID‐19 patient sera samples

For the initial assay characterization, a commercially available panel of 20 COVID patient serum specimens (10 positive SARS‐CoV‐2‐IgG and 10 negative SARS‐CoV‐2‐IgG sera samples), confirmed using the Abbott Architect SARS‐CoV‐2‐IgG assay, were obtained from Boca Biolistics (Cat# C0050‐0001). The samples were heat‐inactivated at 65°C for 30 min to allow safe handling in our BSL2 laboratory, and they were stored in a −80°C freezer until required. For clinical validation, 49 serum samples were obtained under protocol number 1661992‐4 approved by the Institutional Review Board of UC Davis Health (University of California Davis). The positive serum (*n* = 34) (males = 16, out of which 8 had recovered, age range of 25–77 years; females = 18 out of which 8 had recovered, age range of 22–74 years) samples were collected from COVID‐19 patients with confirmed qPCR test results for SARS‐CoV‐2 RNA and confirmed seropositive, while control (*n* = 15) (males = 6, age range of 25–68 years; females = 9, age range of 23–65 years) sera were from healthy individuals and was confirmed seronegative. Gold Standard SARS‐CoV‐2 IgG and IgA ELISAs (Gold Standard Diagnostics, Davis, USA Cat# GSD01‐1029 IgA, GSD01‐1028 IgG) targeting nucleocapsid (N) protein, human SARS‐CoV‐2 spike (Trimer) Ig Total ELISA kit (ThermoFisher Scientific, Cat# BMS2323) targeting spike protein were used to assess sero‐status.

### 4.3 Generation of the Reporter‐Cell (R) component of the DxCell‐Complex

The Jurkat E6‐1 suspension cell line was engineered with lentivirus particles carrying the genetic payload (Figure 1), as detailed previously.^9^ Here, the Receptor (CAbR) domain was replaced with the synthetic sequence from the bacterial Protein A (zz‐domain; GenBank: M74186), previously reported to bind to the Fc region of IgG antibodies.^12^, ^13^ Briefly, the cells were treated with lentivirus in the presence of 8 μg/mL Polybrene. After 48 h, the engineered cells were placed in selection using 0.5 μg/mL of puromycin dihydrochloride. The unmodified parental cell line was also placed under selection as a positive control for determining the minimum lethal concentration of puromycin. Following selection, cells were expanded as required for different assays and frozen using freezing media.

### 4.4 Generation of the Target‐Cell (T) component of the DxCell‐Complex

The HEK293T/17 adherent cells were engineered to stably express viral Sgp, using the PiggyBac Transposon system, as previously described by our group.^10^ Two plasmids were designed with the piggyBac transposon backbone (System Biosciences, Cat # PB510B‐1) to either express the Sgp of SARS‐CoV‐1 (SARS‐CoV‐1‐Sgp; GenBank: AAP13567.1) or of SARS‐CoV‐2 (SARS‐CoV‐2‐Sgp; GenBank: QHD43416.1) on the cell surface. Briefly, a monolayer of HEK293T/17 cells were transfected with both the Transposon plasmid (carrying gene of interest) and Transposase plasmid, in a ratio of 2.5:1, using TransIT‐2020 transfection reagent. After 48 h of transfection, the transfected cells were placed under selection using puromycin dihydrochloride. The unmodified parental HEK293T/17 cell line was also placed under selection as a positive control to determine the minimum lethal concentration of puromycin. The generated stable cell lines were labeled as *SARS‐CoV‐1‐Sgp cells* (HEK293T/17 cells engineered to stably express the Sgp from SARS‐CoV‐1) and *SARS‐CoV‐2‐Sgp cells* (HEK293T/17 cells engineered to stably express the Sgp from SARS‐CoV‐2). The cells were then expanded as required for different assays.

### 4.5 Method of use of DxCell‐Complex for serology test

- With research‐grade antibodies against SARS‐CoV‐1‐Sgp or SARS‐CoV‐2‐Sgp during technology development (Figures 2 and S1). The Reporter Cell (12,500 in 50 μL of complete RPMI media) was incubated with varying concentrations (or as stated) of the Anti‐SARS‐CoV‐1 IgG (S230) or Anti‐SARS‐CoV‐2 IgG monoclonal antibodies for 30 min at 37°C, washed once, and then co‐cultured with 10,000 Target Cells (SARS‐CoV‐1‐Sgp cells or SARS‐CoV‐2‐Sgp cells, respectively) in 100 μL of complete RPMI media per well of a 96‐well plate. As a negative control, the Reporter Cell was coated using a nonspecific Anti‐WNV‐Egp monoclonal antibody and co‐cultured with the same Target Cells. After the specified time in co‐culture, NanoLuc (Nluc) activity in the Reporter Cell was assessed using Nano‐Glo assay kit, following the manufacturer's instructions. Briefly, the Nluc enzyme substrate was diluted in the cell‐lysis buffer provided with the Nano‐Glo and added to the co‐cultures in 96‐well plate for assessing enzyme activity. Following a brief incubation period of 3 min, bioluminescence was read on a microplate reader (Perkin Elmer, Envision Multilabel Plate Reader Model: 2104‐0010A).
- With COVID‐19 patient serum (Figure 3 and Figure 4). For each serum sample, the Reporter Cell (12,500 in 50 μL of complete RPMI media) was incubated with the respective patient serum at different dilutions (or as stated) for 30 min at 37°C, washed once and then co‐cultured with 10,000 (or varying numbers) of target SARS‐CoV‐2‐Sgp cells, in 100 μL of complete RPMI media per well of a 96‐well plate. After the 24 h in co‐culture, NanoLuc (Nluc) activity in the Reporter Cell was assessed using Nano‐Glo assay as described earlier.

### 4.6 Statistical analysis

GraphPad Prism 9.2.0 (GraphPad Software, Inc.) was used to conduct all statistical analyses. The experimental design and logistical models used for each panel in the figures is described further in the Supporting Information.

## AUTHOR CONTRIBUTIONS

Parijat Bhatnagar was the principal investigator, conceptualized the DxCell‐Complex, designed the experimental investigations, and wrote the manuscript. Marvin A. Ssemadaali designed the experimental investigations, performed the assays, and wrote the manuscript. Harold S. Javitz was the co‐investigator and designed the statistical tests. Satya Dandekar advised on experimental designs, while Juan Arredondo, Elise A. Buser, Sherri Newmyer, and Harikrishnan Radhakrishnan conducted experiments. Marvin A. Ssemadaali, Harold S. Javitz, Satya Dandekar, and Parijat Bhatnagar analyzed the results. All authors read and contributed to preparation of the manuscript.

## CONFLICT OF INTEREST STATEMENT

The authors declare no conflicts of interest.

## Supporting information


**Data S1.** Supporting Information.

## Data Availability

All data used to draw conclusions of our work are present in this manuscript and supporting information.
