# Supplementary material for: Genetically engineered pair of cells for serological testing and its application for SARS‐CoV‐2
Source: Bioeng Transl Med. 2023 Mar 24;8(3):e10508. doi: 10.1002/btm2.10508 (PMC10189431; doi:10.1002/btm2.10508)
Supplement: Supplementary file 1 — Data S1. Supporting Information. [file BTM2-8-e10508-s001.pdf]

SUPPORTING INFORMATION

Genetically engineered pair of cells for serological testing and its application for SARS-CoV-2

*Marvin A. Ssemadaali[1], Juan Arredondo[2], Elise A. Buser[2], Sherri Newmyer[1], Harikrishnan Radhakrishnan[1], Harold S. Javitz[3], Satya Dandekar[2], Parijat Bhatnagar[1]**

[1]Biosciences Division, SRI International, Menlo Park, CA 94025

[2]Medical Microbiology and Immunology, University of California Davis, Davis, CA 95616

[3]Education Division, SRI International, Menlo Park, CA 94025

**Parijat.Bhatnagar@sri.com*

Running title: Cell-based COVID-19 serology test

Supplemental statistical analysis. The experimental design and logistical models used for each panel in the figures is described below.

i) *Figure 2 (Development of method for using the DxCell-Complex).* Statistical analyses were based on multiple t-tests using a two-stage linear step-up procedure of Benjamini, Krieger, and Yekutieli with a false discovery rate of 1%. There was no adjustment for multiple comparisons. The S/N is calculated as the ratio of the mean Nluc activity in the Reporter Cell when the DxCell-Complex is incubated with Anti-SARS-CoV-1-Sgp-IgG or Anti-SARS-CoV-2-Sgp-IgG antibodies, divided by the mean Nluc activity in the Reporter Cell when the DxCell-Complex is incubated with Anti-WNV-Egp-IgG antibodies. The error bars extend 1 standard deviation (SD) above and below the mean.

ii) *Figure 2A (Nluc activity in the Reporter Cell is proportional to the concentration of antibodies).* The Nluc activity in the Reporter Cell when stimulated by the target SARS-CoV-1-Sgp-cells was fitted using a semi-log logistic model Y = a + b * log10(X), where X is the

 DOI: 10.1002/BTM2.10508

concentration (ng) of IgG antibodies used to coat the Reporter Cell, a = Y-intercept, and b = Slope.

iii) *Figure 2B (Nluc activity in the Reporter Cell is a function of duration of stimulation).* The Nluc activity in the Anti-SARS-CoV-1-Sgp-coated (or Anti-WNV-Egp-coated) Reporter Cell stimulated by the Target Cells (SARS-CoV-1-Sgp-cells) was fitted using a four-parameter logistic model $Nluc = Nluc_{min} + \{Nluc_{max} – Nluc_{min}\}/\{1 + 10^{\wedge}[b^{*} (log_{10}[Time_{50}] – X)]\}$; where X is the log_{10} of the duration of activation (hours) by the Target Cells, $Nluc_{max}$ is an estimated parameter defining a upper asymptote for Nluc activity, $Nluc_{min}$ is an estimated parameter defining a lower asymptote for Nluc activity, b is a "Hill" parameter defining the slope at the inflection point of the fitted curve, and $Time_{50}$ is an estimated parameter representing the X value corresponding to $(Nluc_{max} - Nluc_{min})/2$.

iv) *Figure 2C and Figure 2D (Nluc activity in the Reporter Cell is proportional to the Target-Cell count).* The Nluc activity in the Reporter Cell when stimulated by the target SARS-CoV-1-Sgp-cells or SARS-CoV-2-Sgp-cells was fitted using a four-parameter logistic model $Nluc = Nluc_{min} + \{Nluc_{max} – Nluc_{min}\}/\{1 + 10^{\wedge}[b^{*} (log_{10}[Target_{50}] – X)]\}$; where X is the log_{10} of the Target-Cell count, $Nluc_{max}$ is an estimated parameter defining a upper asymptote for Nluc activity, $Nluc_{min}$ is an estimated parameter defining a lower asymptote for Nluc activity, b is a "Hill" parameter defining the slope at the inflection point of the fitted curve, and $Target_{50}$ is an estimated parameter representing the X value corresponding to $(Nluc_{max} – Nluc_{min})/2$. **Figure 2C** shows results with Anti-SARS-CoV-1-IgG (S230) while **Figure 2D** shows results with Anti-SARS-CoV-2-IgG (Anti-WNV-IgG as negative control in both experiments).

v) *Figure 3 (Characterization of the DxCell-Complex for serology test using a commercial serum panel).* Statistical analysis was based on an unpaired two-tailed student's t-test with common variance and the p-value of <0.05 was considered statistically significant. Statistical analysis for **Figure 3B** was based on multiple t-tests using a two-stage linear step-up procedure

of Benjamini, Krieger, and Yekutieli with a false discovery rate of 1%. The error bars extend 1 SD above and below the mean. Analysis in **Figure 3A, 3C and 3D** used the same data values.

vi) Figure 3A (DxCell-Complex differentiates between positive patient sera and negative sera samples). An estimation plot (confidence interval of 95%) shows the difference between positive patient sera and negative sera samples, using an unpaired two-tailed student's t-test, assuming Gaussian distribution and that both sample means have the same standard deviations.

vii) Figure 3B (Specificity of the DxCell-Complex in differentiating sera samples with varying IgG antibody titers). The Nluc activity in the serum-coated Reporter Cell stimulated by the target SARS-CoV-2-Sgp-cells was fitted using a straight-line equation $Y = a + b * \log_{10}(X)$; where X is the Target-Cell count used to activate the Reporter Cell, a = Y-intercept, and b = Slope.

viii) Figure 3C (Receiver operator characteristics [ROC] curve analysis). The ROC curve analysis was performed using the Wilson/Brown method at 95% Confidence Interval, and results on the ROC curve are expressed as percentages. Abbott Architect SARS-CoV-2-IgG assay was used to classify patient samples. Commercially available panel of 20 COVID patient serum specimens (10 positive SARS-CoV-2-IgG and 10 negative SARS-CoV-2-IgG sera samples), confirmed using the Abbott Architect SARS-CoV-2-IgG assay, were used.

ix) Figure 3D (Correlation between the DxCell-Complex serology test and the gold standard test). Simple linear regression was used to plot the goodness of fit between the two assays and determine the R^2 statistic and the Pearson's correlation coefficient, r. The p-value of the F-statistic was used to determine statistical significance of the regression. X-axis indicates Abbott Architect SARS-CoV-2-IgG assay Index (S/C), where S is the chemiluminescent signal from the specimen, and C is the system generated mean chemiluminescent signal from three calibrator replicates. It indicates the amount of antibodies to SARS-CoV-2 as detected by the assay.

x) *Figure 4A (Clinical validation of the DxCell-Complex for detecting COVID-19 IgG antibodies in patients).* A scatterplot shows the difference between COVID-19 patient sera and control sera samples, using an unpaired two-tailed student's t-test, assuming a Gaussian distribution with a Welch's correction for degrees of freedom since standard deviations are not assumed equal.

xi) *Figure 4B (Receiver operator characteristics [ROC] curve analysis).* The ROC curve analysis was performed using the Wilson/Brown method at 95% Confidence Interval, and results on the ROC curve are expressed as percentages. RT-qPCR results were used to determine clinical status and classify patient serum. Serum samples were collected from 34 individuals who were diagnosed for SARS-CoV-2 infection (18 were ill at draw, 16 had recovered) based on detection of SARS-CoV-2 RNA as well as seropositivity and 15 healthy seronegative controls.

xii) *Figure S1 (Sensitivity of the DxCell-Complex while using serially diluted sera).* The differences between the positive and negative sera in the multiple box plots was determined using the Holm-Sidak method, assuming that all sample groups within each dilution had similar standard deviations. A threshold alpha value of 0.1 for adjusted P value comparisons was used, which reduces the probability of a false positive across all comparisons to 10%.

xiii) *Table S1 and Table S2 (Contingency table analysis for human COVID-19 sera samples).* Confidence intervals for the specificities, sensitivities, and predictive values were calculated using the Wilson/Brown method. The p-values were calculated using the Fisher's exact t-test. **Table S1** shows results using a commercial serum panel while **Table S2** shows results using clinical samples from UC Davis Health.

SUPPLEMENTARY FIGURES AND TABLES

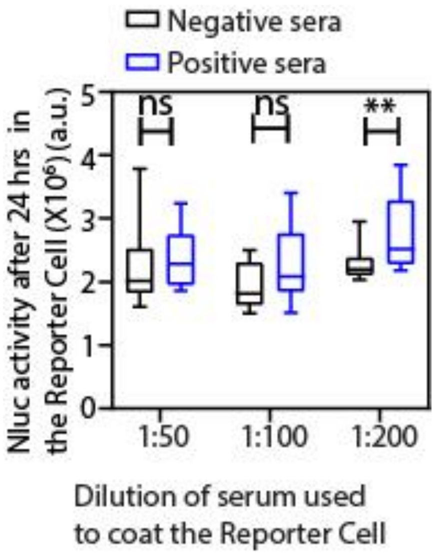

Figure S1. Sensitivity of the DxCell-Complex using different sera dilutions. The Nluc activity in the Reporter Cell (12,500 cells) varied with respect to serum dilutions. SARS-CoV-2-Sgp-cells (10,000 cells) were used. Each data bar represents Nluc activity for observations measured using n = 10; error bars indicate ±1 SD. The text “ns” indicates P value was not below the threshold value of 0.1; ** indicates P value was below the threshold value of 0.1 (i.e., there is less than a 10% chance that any comparison labeled ** is a false positive).

 DOI: 10.1002/BTM2.10508

Table S1. Contingency table analysis to determine the accuracy of the DxCell-Complex, using the Wilson-Brown method; Abbott Architect IgG assay used as the gold standard.

Data analyzed	***COVID-19 IgG positive***	***COVID-19 IgG negative***	***Total***
DxCell-Complex Positive	8	3	11
DxCell-Complex Negative	2	7	9
Total	10	10	20

Effect size	**Value**	**95% CI**
Sensitivity	0.8	0.4902 to 0.9645
Specificity	0.7	0.3968 to 0.8922
Positive Predictive Value	0.7273	0.4344 to 0.9025
Negative Predictive Value	0.7778	0.4526 to 0.9605
Likelihood Ratio	2.667	

Table S2. Contingency table analysis to determine the accuracy of the DxCell-Complex, using the Wilson-Brown method; RT-qPCR used to determine clinical status of patients.

Data analyzed	***COVID-19 positive***	***COVID-19 negative***	***Total***
DxCell-Complex Positive	33	1	34
DxCell-Complex Negative	1	14	15
Total	34	15	49

Effect size	**Value**	**95% CI**
Sensitivity	0.9706	0.8508 to 0.9985
Specificity	0.9333	0.7018 to 0.9966
Positive Predictive Value	0.9706	0.8508 to 0.9985
Negative Predictive Value	0.9333	0.7018 to 0.9966
Likelihood Ratio	14.56	
